# The early impact of the COVID-19 pandemic on international fellows

**DOI:** 10.62675/2965-2774.20260336

**Published:** 2026-05-20

**Authors:** Michael Chaim Sklar, Joanna Bouez, Hannah C. Joo, Nawid Sayed, Christie Lee, Alpna Munshi, Umberin Najeeb, Sangeeta Mehta, Alya Kamani, Mika Hamilton, Laveena Munshi

**Affiliations:** 1 University of Toronto St Michael's Hospital Department of Anaesthesia, Unity Health Toronto Ontario Canada Interdepartmental Division of Critical Care Medicine, Department of Anaesthesia, Unity Health, St Michael's Hospital, University of Toronto - Toronto, Ontario, Canada.; 2 Queens University Department of Medicine Kingston Ontario Canada Department of Medicine, Queens University – Kingston, Ontario, Canada.; 3 Interdepartmental Division of Critical Care Medicine Department of Medicine, Sinai Health System Toronto Ontario Canada Department of Medicine, Sinai Health System; Interdepartmental Division of Critical Care Medicine, University of Toronto - Toronto, Ontario, Canada.; 4 University of Toronto Department of Psychiatry Toronto Ontario Canada Department of Psychiatry - University of Toronto - Toronto, Ontario, Canada.; 5 Department of Medicine, Sunnybrook Health Sciences Center Toronto Ontario Canada Department of Medicine, Sunnybrook Health Sciences Center - Toronto, Ontario, Canada.; 6 Department of Critical Care Medicine, Sunnybrook Health Sciences Center Toronto Ontario Canada Department of Critical Care Medicine, Sunnybrook Health Sciences Center - Toronto, Ontario, Canada.; 7 Sinai Health System/University Health Network Interdepartmental Division of Critical Care Medicine Toronto Ontario Canada Interdepartmental Division of Critical Care Medicine, Sinai Health System/University Health Network, University of Toronto - Toronto, Ontario, Canada.

## INTRODUCTION

In March 2020, drastic public health measures such as travel restrictions and social distancing were implemented to curb the spread of coronavirus disease 2019 (COVID-19).^(1)^ While the psychological impacts of these measures on the general population and healthcare workers have been well studied,^(2–7)^ international physician trainees (fellows) faced additional challenges. We evaluated the impact of the COVID-19 pandemic on international fellows at a Canadian academic institution, highlighting institutional gaps and opportunities to strengthen preparedness.

## METHODS

A prospective electronic survey was distributed to 237 international fellows in general internal medicine and subspecialties at the University of Toronto (June to July 2020). The survey assessed the effects of COVID-19 measures on work, training objectives, well-being, and support systems, including perceived institutional gaps.

Likert scales (zero to five) measured satisfaction with support, stress, the impact of wellness on patient care, and degree of worry. Open-ended responses allowed suggestions for improvements. Surveys were administered via SurveyMonkey with three reminders. Institutional research ethics approval was obtained for the study (REB# MSH REB 20-0128-E). Consent was implied by survey completion. Results were summarized descriptively.

## RESULTS

Eighty-eight fellows (37%) responded, most male (55%), married or common-law (72%), and over half lived with dependents (53%). Fellows originated mainly from the Middle East (20%), South/East Asia (20%), and Europe (18%). Fourteen specialties were represented, with critical care (30%) and oncology (15%) being the most common (Table 1).

**Table 1:** Demographic details and clinical characteristics of surveyed international fellows at the University of Toronto

Variables		n (%)
Gender		
	Male	48 (55)
	Female	38 (43)
	Prefer not to say	2 (2)
Age (years)		
	26 - 30	4 (5)
	31 - 35	40 (45)
	36 - 40	28 (32)
	41 - 45	13 (15)
	46 - 50	3 (3)
Marital status		
	Single	23 (26)
	Married/common-law	63 (72)
	Divorced/Separated	2 (2)
Number of children/dependents		
	0	41 (4)
	1	23 (26)
	2	16 (18)
	3	5 (6)
	4	3 (3)
Cohabitants		
	Partner	50 (57)
	Children	37 (42)
	None	28 (32)
	Relatives	4 (5)
	Roommate(s)	3 (3)
	Parents	3 (3)
Location of origin		
	North America	4 (5)
	Central America	4 (5)
	South America	13 (15)
	Europe	16 (18)
	South/East Asia	17 (20)
	Middle East	17 (20)
	Africa	3 (3)
	Australia	9 (10)
	Prefer not to say	4 (5)
Specialty program		
	Critical care medicine	26 (30)
	Oncology	13 (15)
	Nephrology	10 (11)
	Gastroenterology	10 (11)
	Hematology	9 (10)
	Transplant medicine	8 (9)
	Rheumatology	7 (8)
	Infectious disease	3 (3)
	Neurology	2 (2)
	Palliative care	2 (2)
	Hospital medicine	1 (1)
	Cardiology	1 (1)
	Research fellowship	1 (1)
	Toxicology	1 (1)
Provide in-house on-call services		
	Yes	56 (69)
	No	25 (31)
Contact with COVID-19 patients		
	Aerosol generating procedures	26 (32)
	No aerosol-generating procedures	22 (27)
Do not provide care for COVID-19 patients		
	Pandemic impact on workload	32 (40)
	Increase	20 (25)
	Decrease	29 (36)
	No change	33 (41)

Working hours varied: 25% reported increases, 36% decreases, and 39% no change. Training objectives were disrupted for 70%. Of these, 60% reported suspended research, 56% reduced patient exposure, 50% a limited case mix, and 46% training focused on COVID-19. Nearly half (49%) would have preferred to be in their home country, and 51% considered returning. Of the sample, 38% reported altered training plans, extending training due to travel restrictions or returning home early.

**Figure 1:**
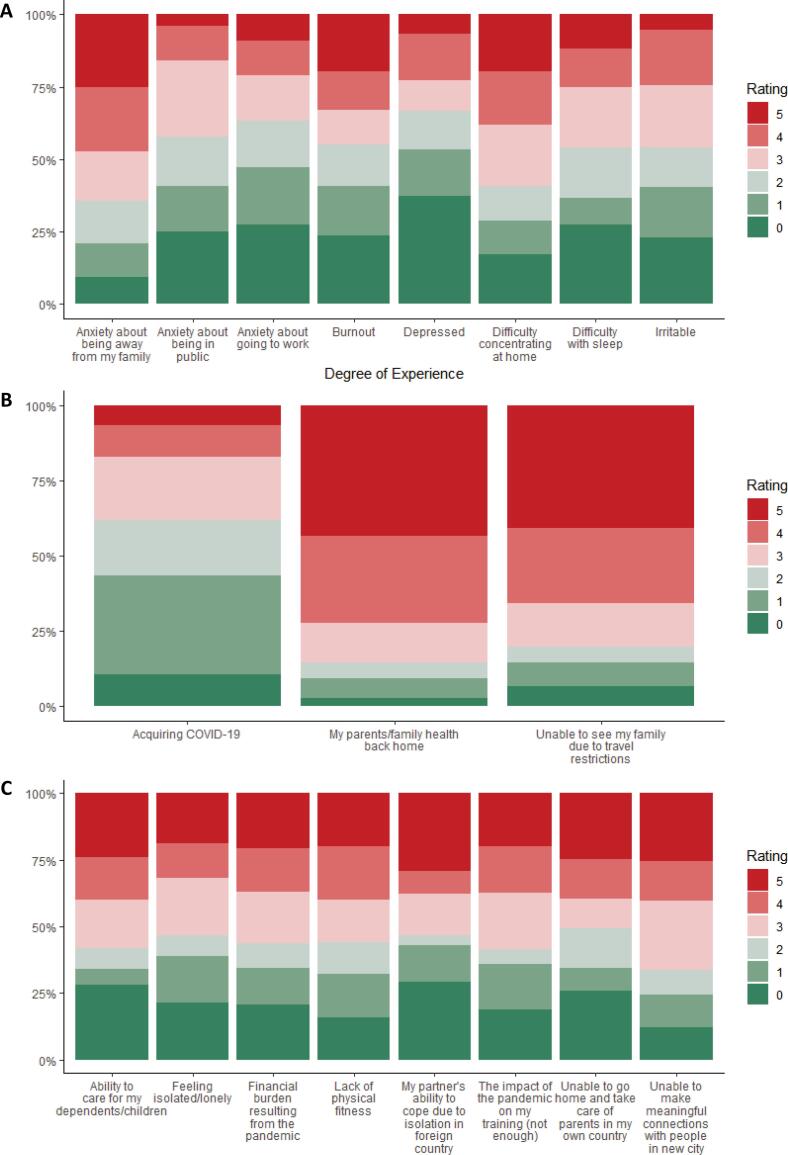
Psychological symptoms, stressors, and challenges reported by international fellows during the early COVID-19 pandemic (n = 76).

Psychological strain was substantial. Moderate to significant anxiety was reported by 64%, sleeping difficulties by 46%, difficulty concentrating by 59%, irritability by 45%, depression by 33%, and burnout by 45%. Half believed their wellness affected patient care; one-quarter reported "a fair amount" or "a great deal" of impact (Figure 1).

Proportion of international fellows (n = 76) reporting (A) mental health symptoms, (B) key pandemic-related stressors, and (C) challenges specific to international fellows. Respondents rated severity or level of concern on a 6-point Likert scale (0 = not at all; 5 = very significant).

COVID-19 educational resources were the most highly rated support (89% adequate), yet 41% cited not knowing what supports were available as a barrier. Key stressors included worry about family health abroad (86%) and inability to visit family (80%).

Qualitative responses highlighted isolation from family, challenges with childcare, and disappointment at being unable to explore Canada. Some described the experience as a "learning opportunity", noting that Canada's response was stronger than that of their home countries. Suggested support included communication with supervisors, peer meetings, workload reductions, financial aid, and childcare options, highlighting areas for institutional strengthening.

## DISCUSSION

International fellows demonstrated heightened vulnerability during the pandemic, largely due to distance from family and limited local support. The primary burden was psychological rather than workload-related, with high rates of anxiety, burnout, and depression - findings consistent with broader literature on healthcare worker distress.^(3–6)^

The perception that wellness negatively influences patient care underscores the need to prioritize trainee support. Fellows’ suggestions - including structured communication, peer connection, childcare support, and supervisor check-ins - align with World Health Organization (WHO) recommendations^(7)^ and prior calls for institutional strategies to support international trainees.^(8–10)^

Five years later, these observations remain relevant, as training programs refine crisis-responsive educational frameworks and wellness pathways. Embedding predictable communication structures, check-ins, and accessible supports into routine programming may strengthen institutional resilience and better support international trainees during future disruptions.

## CONCLUSION

International fellows experienced significant psychosocial strain during the early phase of the COVID-19 pandemic. Integrating targeted supports such as structured communication, social integration, childcare assistance, and accessible wellness resources into fellowship programs can strengthen institutional preparedness and better protect international trainees in future crises.

## Data Availability

The contents underlying the research text are included in the manuscript.
